# Nature-Identical Compounds and Organic Acids Ameliorate and Prevent the Damages Induced by an Inflammatory Challenge in Caco-2 Cell Culture

**DOI:** 10.3390/molecules25184296

**Published:** 2020-09-19

**Authors:** Andrea Toschi, Barbara Rossi, Benedetta Tugnoli, Andrea Piva, Ester Grilli

**Affiliations:** 1 Department of Veterinary Medical Sciences, University of Bologna, Via Tolara di Sopra n. 50, 40064 Ozzano dell’Emilia (BO), Italy; andrea.toschi5@unibo.it (A.T.); andrea.piva@unibo.it (A.P.); 2 Vetagro S.p.A., via Porro 2, 42124 Reggio Emilia, Italy; barbara.rossi@vetagro.com (B.R.); benedetta.tugnoli@vetagro.com (B.T.); 3 Vetagro Inc., 116W. Jackson Blvd., Suite #320, Chicago, IL 60604, USA

**Keywords:** organic acid, nature-identical compound, barrier function, Caco-2 cells, inflammatory response

## Abstract

Bioactive compounds, such as organic acids (OA) and nature-identical compounds (NIC), can exert a role in the protection of intestinal mucosa functionality due to their biological properties. The aim of this study was to understand the role of 2 OA (citric and sorbic acid) and 2 NIC (thymol and vanillin), alone or combined in a blend (OA + NIC), on intestinal barrier functionality, either during homeostatic condition or during an inflammatory challenge performed with pro-inflammatory cytokines and lipopolysaccharides (LPS). The study was performed on the human epithelial cell line Caco-2, a well-known model of the intestinal epithelial barrier. The results showed how OA and NIC alone can improve transepithelial electrical resistance (TEER) and mRNA levels of tight junction (TJ) components, but OA + NIC showed stronger efficacy compared to the single molecules. When an inflammatory challenge occurred, OA + NIC blend was able both to ameliorate, and prevent, damage caused by the pro-inflammatory stimulus, reducing or preventing the drop in TEER and improving the TJ mRNA expression. The data support the role of OA + NIC in modulating gut barrier functionality and reducing the negative effects of inflammation in intestinal epithelial cells, thereby supporting the gut barrier functionality.

## 1. Introduction

The intestinal mucosa is composed of a single layer of epithelial cells, connected to each other through inter-epithelial junctions, and it is the first line of physical defense against harmful agents [1]. The maintenance of this barrier function contributes to the homeostasis and health of the animals, since barrier dysfunction might play a role in gut inflammation and disease, thereby reducing growth performance [2]. Tight junctions (TJ) are one of the junctional multiprotein complexes which help the adhesion of adjacent epithelial cells, functioning as the gate keeper to control the diffusion of solutes, regulating ion transport, blocking of macromolecules and controlling the selective transport of nutrients [3]. Dysregulation of TJ enables harmful substances to translocate, resulting in a damaged tissue and inflammation. More than 30 structural and functional proteins constitute the TJ complex, among them zonula occludens-1 (ZO-1) and occludin are two crucial proteins to maintain the structural function of TJ [4]. Moreover, recent outcomes have highlighted the role of transient potential receptor vanilloid (TRPV) channels in modulating inflammation, while their precise role is still under investigation. In fact, TRPV channels are reported both as pro-inflammatory and as anti-inflammatory [5,6]. When intestinal barrier failure occurs, the concentration of pro-inflammatory cytokines, produced by the lamina propria, increases [7], which negatively influences intestinal integrity, and leading to a reduction in intestinal functionality [8,9]. For these reasons, it is important to develop strategies to prevent intestinal barrier failure, supporting animal healthy growth (especially in the early stages of life like weaning). In this context, bioactive compounds such as organic acids (OA) and nature-identical compounds (NIC) can exert a role in the maintenance of intestinal functionality, due to their numerous biological properties [10,11,12]. In particular, our research group [13] reported the beneficial effects of a blend of OA (citric and sorbic acid) and NIC (thymol and vanillin), on porcine intestinal barrier functionality, through a stimulation of the local immune response. Moreover, the results from that study demonstrated how these compounds, together, can improve the intestinal barrier functionality on Caco-2 cells, by increasing transepithelial electrical resistance (TEER) and reducing intestinal permeability to dextran (paracellular permeability, PCP). Nevertheless, little is known about their actual mode of action. While, understanding the mechanisms underlying the effects of these OA and NIC, and more in general of bioactive compounds, is critical to their safe and effective application in animal nutrition. So far, the alleged mechanism of action of OA and NIC as growth enhancers was mostly connected to their well-documented antimicrobial properties, thereby implicating a possible role of these compounds in modulating the intestinal microflora. On the other side, recent clues suggest that these compounds have a direct role in priming and boosting the host immune response [14].

In the present study, we intended to help elucidate the mechanism of action of citric and sorbic acid, thymol, and vanillin in preventing or improving intestinal barrier failure. To exclude any possible effect/interference mediated by the microflora, we performed in vitro experiments and used Caco-2 cells as a model to study the intestinal epithelium. As in vitro model, Caco-2 cells is frequently used due to its morphological, ultrastructural, and biochemical similarities with small intestinal epithelial cells for both pigs and humans [15]. Two sets of experiments were performed, one to assess the role of these molecules in “healthy conditions” (Experiment 1) and the second set to understand their role in “challenged conditions” that potentially mimic stressful events like inflammation at weaning (Experiment 2).

## 2. Results

### 2.1. Experiment 1—Dose Response

Experiment 1 was performed to investigate the potential effect of OA and NIC, alone or combined in a blend, on intestinal epithelium. The results are divided as follows: citric acid, sorbic acid, thymol, vanillin, and the blend of OA + NIC. These molecules were tested at increasing concentrations to determine viability, TEER, PCP, and gene expression were evaluated.

#### 2.1.1. Citric Acid

**Figure 1:**
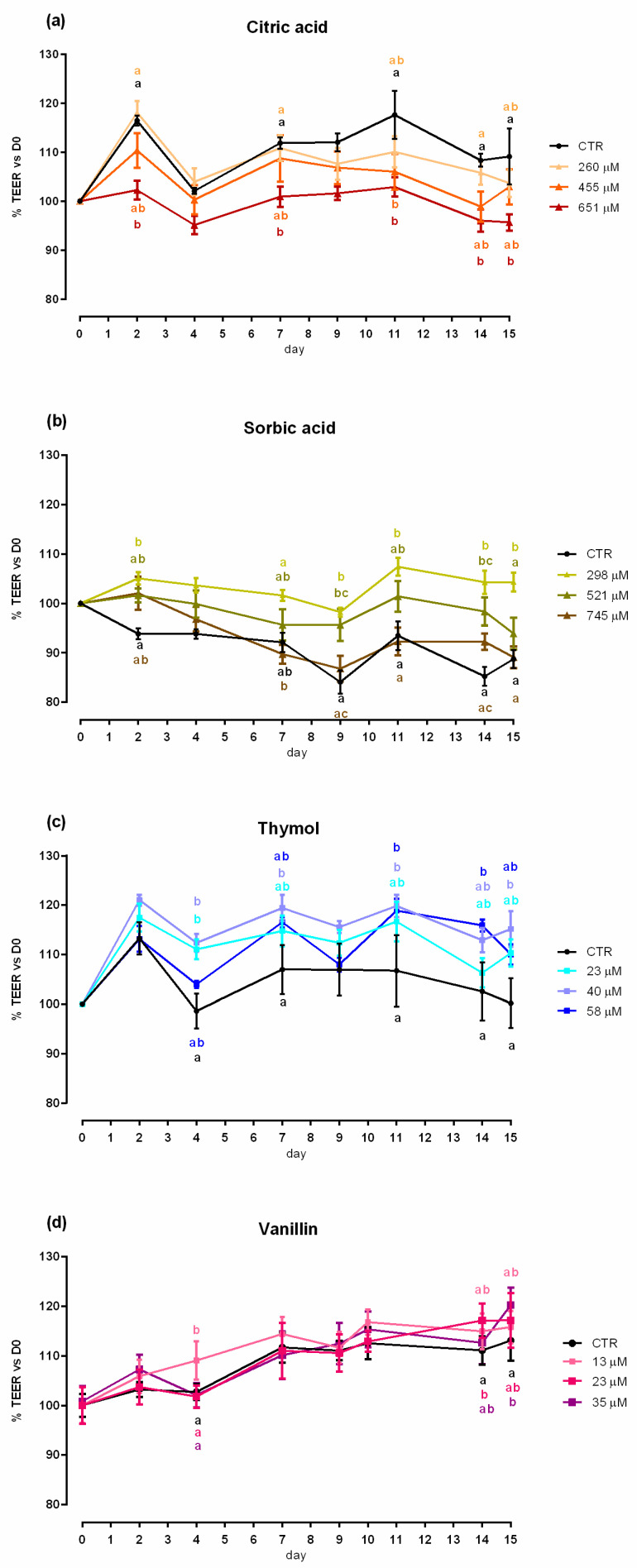
TEER of Caco-2 cells cultured with single OA and NIC. Data in the graph are represented as percentage over the initial TEER value and given as means (*n* = 6) ± SEM, represented by vertical bars. Means with different letters indicate statistical significance with *p* < 0.05 (a, b); means with at least one common letter are not significantly different (ab), colors of the letters refer to the different treatments. TEER = transepithelial electrical resistance (Ω·cm^2^). (**a**) Cells treated with citric acid; CTR = control group; 260 = treated group with 260 μM of citric acid; 455 = treated group with 455 μM of citric acid; 651 = treated group with 651 μM of citric acid. (**b**) Cells treated with sorbic acid; CTR = control group; 298 = treated group with 298 μM of sorbic acid; 521 = treated group with 521 μM of sorbic acid; 745 = treated group with 745 μM of sorbic acid. (**c**) Cells treated with thymol; CTR = control group; 23 = treated group with 23 μM of thymol; 40 = treated group with 40 μM of thymol; 58 = treated group with 58 μM of thymol. (**d**) Cells treated with vanillin; CTR = control group; 13 = treated group with 13 μM of vanillin; 23 = treated group with 23 μM of vanillin; 35 = treated group with 35 μM of vanillin.

**Figure 2:**
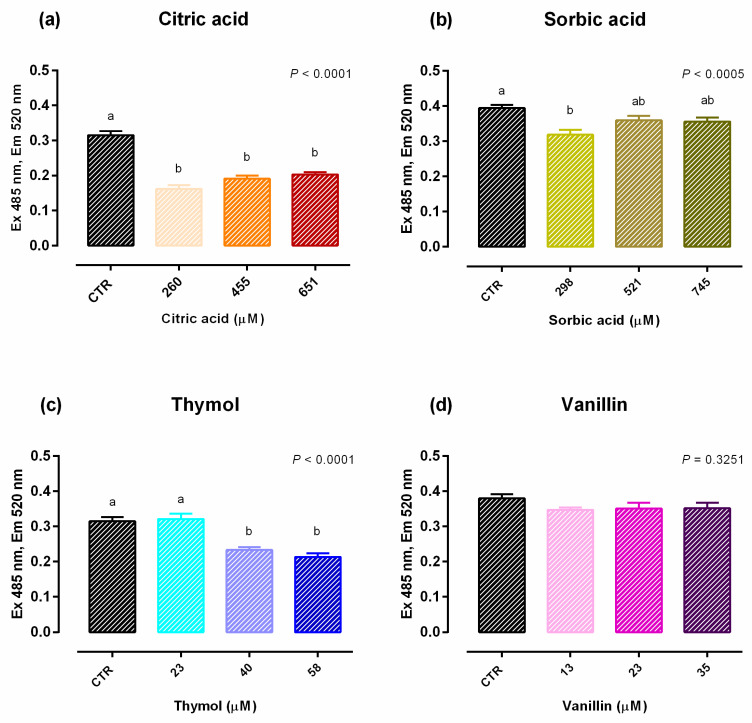
PCP of Caco-2 cells grown with single OA and NIC. Data in the graph are means (*n* = 6) ± SEM represented by vertical bars. Means with different letters indicate statistical significance with *p* < 0.05 (a, b); means with at least one common letter are not significantly different (ab). PCP = paracellular permeability (excitation 485 nm, emission 520 nm). (**a**) Cells treated with citric acid; CTR = control group; 260 = treated group with 260 μM of citric acid; 455 = treated group with 455 μM of citric acid; 651 = treated group with 651 μM of citric acid. (**b**) Cells treated with sorbic acid; CTR = control group; 298 = treated group with 298 μM of sorbic acid; 521 = treated group with 521 μM of sorbic acid; 745 = treated group with 745 μM of sorbic acid. (**c**) Cells treated with thymol; CTR = control group; 23 = treated group with 23 μM of thymol; 40 = treated group with 40 μM of thymol; 58 = treated group with 58 μM of thymol. (**d**) Cells treated with vanillin; CTR = control group; 13 = treated group with 13 μM of vanillin; 23 = treated group with 23 μM of vanillin; 35 = treated group with 35 μM of vanillin.

**Figure 3:**
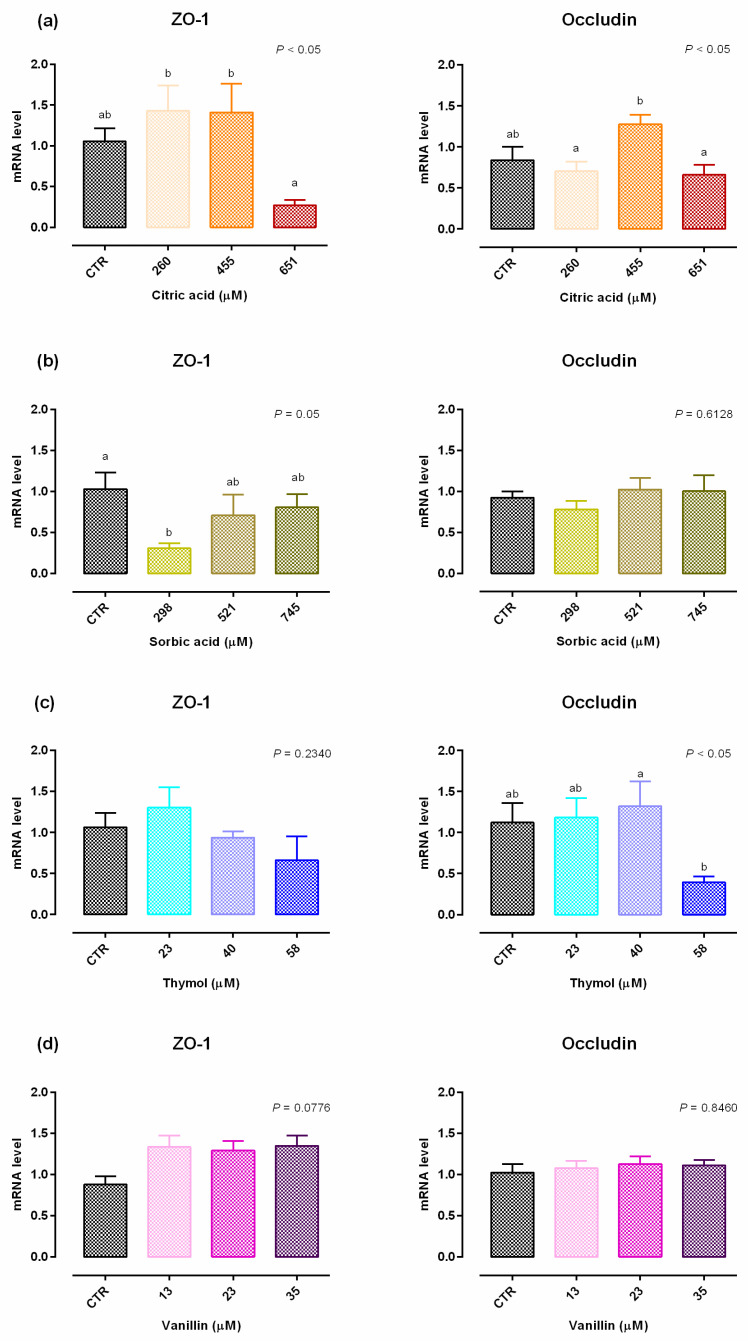
Gene expression in Caco-2 cells after 15 days of treatment with single OA and NIC. Values are least square means (*n* = 6) ± SEM represented by vertical bars. A modification of the 2^–ΔΔCT^ method [16] was used to analyze the relative expression (fold changes), calculated relative to the control group. Means with different letters indicate statistical significance with *p* < 0.05 (a, b); means with at least one common letter are not significantly different (ab). (**a**) Cells treated with citric acid; CTR = control group; 260 = treated group with 260 μM of citric acid; 455 = treated group with 455 μM of citric acid; 651 = treated group with 651 μM of citric acid. (**b**) Cells treated with sorbic acid; CTR = control group; 298 = treated group with 298 μM of sorbic acid; 521 = treated group with 521 μM of sorbic acid; 745 = treated group with 745 μM of sorbic acid. (**c**) Cells treated with thymol; CTR = control group; 23 = treated group with 23 μM of thymol; 40 = treated group with 40 μM of thymol; 58 = treated group with 58 μM of thymol. (**d**) Cells treated with vanillin; CTR = control group; 13 = treated group with 13 μM of vanillin; 23 = treated group with 23 μM of vanillin; 35 = treated group with 35 μM of vanillin. ZO-1 = zonula occludens.

An increase in viability was reported with 260 µM of citric acid after 24 h of treatment, while after 7 days a significant reduction of the vitality (up to 10%) was reported with 651 µM of citric acid (Figure S1). Compared to control (CTR), 651 µM negatively impacted TEER on days 2, 7, 11, 14 and 15 (Figure 1). A reduction in PCP was reported for any concentration of citric acid (Figure 2). The results regarding gene expression are reported in Figure 3. Compared to CTR, citric acid did not affect the expression of TJ proteins at any concentration.

#### 2.1.2. Sorbic Acid

Data obtained from viability assay reported a dose-dependent increase after 24 h of treatment with sorbic acid, starting from the lowest level of inclusion. After 7 days cell viability was significantly decreased in a dose dependent manner, compared to CTR (Figure S1). Figure 1 shows the results of TEER. Compared to CTR, TEER was consistently increased by the supplementation of 298 μM of sorbic acid on day 7 and thereafter. Treatment with 521 μM of sorbic acid significantly increased TEER at day 9 and 14, compared to CTR. Sorbic acid at 745 μM did not differ from CTR. Consistently with TEER, PCP was significantly reduced by the supplementation of 298 μM of sorbic acid (Figure 2). Results about gene expression are reported in Figure 3. Compared to CTR mRNA level of ZO-1 was lower in the group treated with 298 µM. No differences were reported for occludin.

#### 2.1.3. Thymol

Viability of Caco-2 cells was not affected by thymol at any concentration or time point (Figure S1). Compared to CTR, 23 μM of thymol increased TEER at day 4, while 40 μM increased TEER starting from day 4 and constantly until day 15. Thymol at 58 µM increased TEER at day 11 and 14, compared to CTR (Figure 1). The PCP was significantly reduced by the supplementation of 40 μM and 58 μM of thymol (Figure 2). Results of gene expression analysis of TJ markers are represented in Figure 3. The mRNA level of ZO-1 and occludin showed no difference between CTR and treated groups.

#### 2.1.4. Vanillin

No variation in terms of viability was observed at any of the tested doses (Figure S1). Compared to CTR, at day 4 an increase in TEER was reported for cells supplemented with 13 µM of vanillin, while an increase for group treated with 23 µM was registered at day 14, and at day 15 for 35 µM (Figure 1). No variations of PCP were reported (Figure 2). A trend of increase in mRNA levels of ZO-1 was reported (*p* = 0.07) (Figure 3).

#### 2.1.5. Blend of Organic Acids and Nature-Identical Compounds

**Figure 4:**
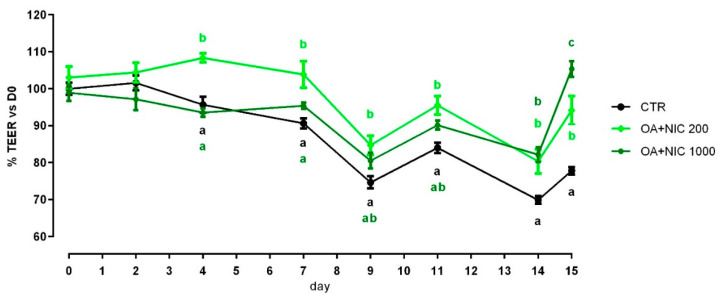
TEER of Caco-2 cells cultured with OA + NIC. Data in the graph are represented as percentage over the initial TEER value and given as means (*n* = 6) ± SEM, represented by vertical bars. Means with different letters indicate statistical significance with *p* < 0.05 (a, b); means with at least one common letter are not significantly different (ab), colors of the letters refer to the different treatments. TEER = transepithelial electrical resistance (Ω·cm^2^); CTR = control group; OA + NIC 200 = treated group with 200 ppm of OA + NIC, composed from 260 µM of citric acid, 298 µM of sorbic acid, 23 µM of thymol, and 13 µM of vanillin; OA + NIC 1000 = treated group with 1000 ppm of OA + NIC, composed from 1300 µM of citric acid, 1490 µM of sorbic acid, 110 µM of thymol, and 70 µM of vanillin.

**Figure 5:**
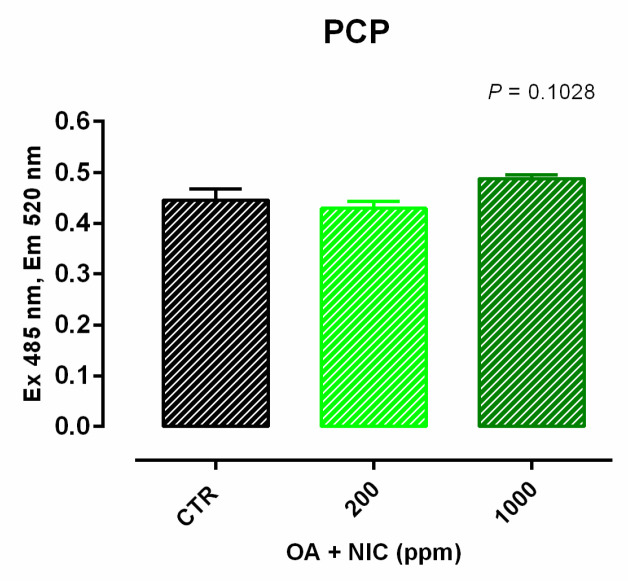
PCP of Caco-2 cells after 15 days of treatment with OA + NIC. Data in the graph are means (*n* = 6) ± SEM represented by vertical bars. Means with different letters indicate statistical significance with *p* < 0.05 (a, b); means with at least one common letter are not significantly different (ab). PCP = paracellular permeability (excitation 485 nm, emission 520 nm). CTR = control group; OA + NIC 200 = treated group with 200 ppm of OA + NIC, composed from 260 µM of citric acid, 298 µM of sorbic acid, 23 µM of thymol, and 13 µM of vanillin; OA + NIC 1000 = treated group with 1000 ppm of OA + NIC, composed from 1300 µM of citric acid, 1490 µM of sorbic acid, 110 µM of thymol, and 70 µM of vanillin.

**Figure 6:**
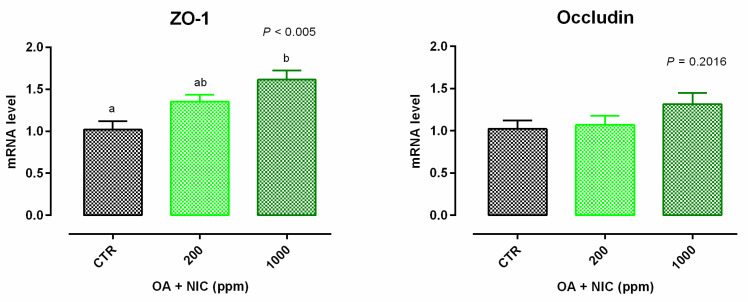
Gene expression in Caco-2 cells after 15 days of treatment with OA + NIC. Values are least square means (*n* = 6) ± SEM represented by vertical bars. A modification of the 2^–ΔΔCT^ method [16] was used to analyze the relative expression (fold changes), calculated relative to the control group. Means with different letters indicate statistical significance with *p* < 0.05 (a, b); means with at least one common letter are not significantly different (ab). CTR = control group; OA + NIC 200 = treated group with 200 ppm of OA + NIC, composed from 260 µM of citric acid, 298 µM of sorbic acid, 23 µM of thymol, and 13 µM of vanillin; OA + NIC 1000 = treated group with 1000 ppm of OA + NIC, composed from 1300 µM of citric acid, 1490 µM of sorbic acid, 110 µM of thymol, and 70 µM of vanillin; ZO-1 = zonula occludens 1.

**Figure 7:**
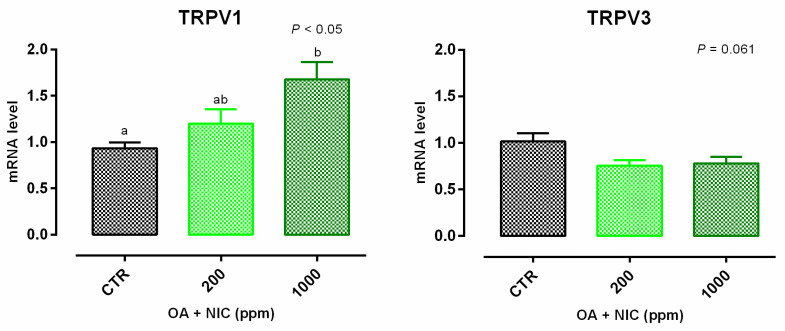
Gene expression in Caco-2 cells after 15 days of treatment with OA + NIC. Values are least square means (*n* = 6) ± SEM represented by vertical bars. A modification of the 2^–ΔΔCT^ method [16] was used to analyze the relative expression (fold changes), calculated relative to the control group. Means with different letters indicate statistical significance with *p* < 0.05 (a, b); means with at least one common letter are not significantly different (ab). CTR = control group; OA + NIC 200 = treated group with 200 ppm of OA + NIC, composed from 260 µM of citric acid, 298 µM of sorbic acid, 23 µM of thymol, and 13 µM of vanillin; OA + NIC 1000 = treated group with 1000 ppm of OA + NIC, composed from 1300 µM of citric acid, 1490 µM of sorbic acid, 110 µM of thymol, and 70 µM of vanillin; TRPV1 = Transient Receptor Potential Vanilloid 1; TRPV3 = Transient Receptor Potential Vanilloid 3.

Cell viability was increased after 24 h and 7 days of treatment with 300 ppm and 700 ppm (Figure S2). TEER data are reported in Figure 4. Compared to CTR, TEER was increased by the supplementation of OA + NIC at 200 ppm (OA + NIC 200), starting from day 4, and the increment remained significant throughout the study, until day 15. TEER was increased also by the supplementation of OA + NIC at 1000 ppm (OA + NIC 1000) at day 14 and day 15 (compared to CTR). About TJ, ZO-1 was increased by OA + NIC 1000, compared to CTR, while OA + NIC 200 showed an intermediate value. No variation of PCP was reported (Figure 5). Occludin mRNA level showed a numerical increase (*p* = 0.20) in the group treated with OA + NIC 1000, compared to control (Figure 6). Moreover, TRPV1 (Transient Receptor Potential Vanilloid 1) was increased by OA + NIC 1000 compared to CTR, while OA + NIC 200 showed an intermediate value. The mRNA level of TRPV3 (Transient Receptor Potential Vanilloid 3) was tendentially reduced by the treatments (*p* = 0.06) (Figure 7).

### 2.2. Experiment 2—Inflammatory Challenge

The purpose of experiment 2 was to assess the role of the blend of OA + NIC in “challenging conditions” induced by the addition of a cocktail of inflammatory cytokines and lipopolysaccharides (LPS). The results of the ameliorative or preventing potential of OA + NIC against the inflammatory challenge were divided in 2 subsections. The therapeutic approach was defined as the ability of the blend to re-establish or ameliorate the parameters evaluated after the challenge. The preventive approach was defined as the capacity of OA + NIC to help cells to prevent the negative effects induced by the inflammatory stimuli.

#### 2.2.1. Therapeutic Approach

**Figure 8:**
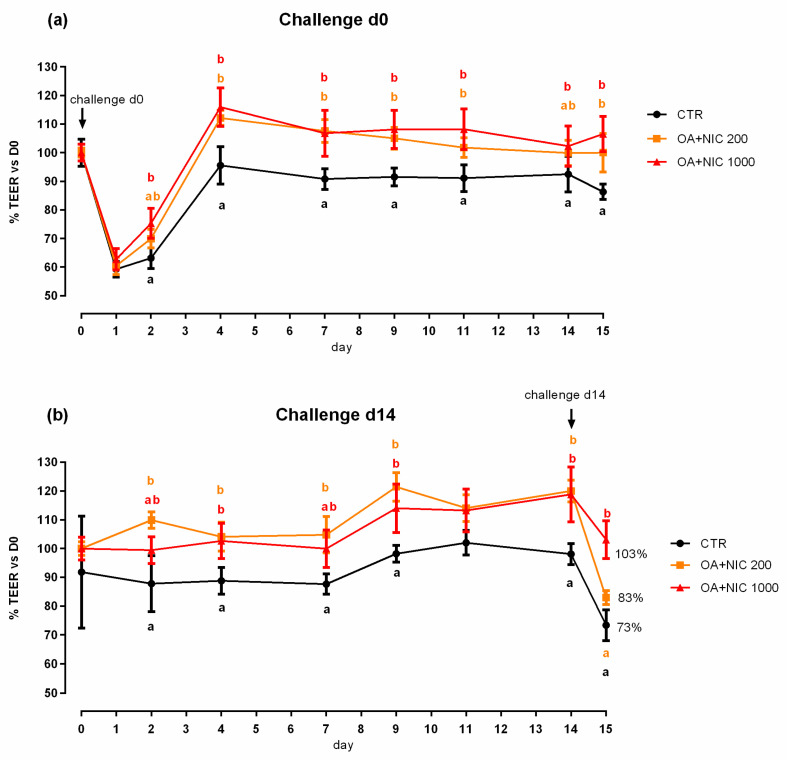
TEER of Caco-2 cells cultured with OA + NIC and subjected to an inflammatory challenge. Data in the graph are represented as percentage of the initial TEER value and given as means (*n* = 6) ± SEM, represented by vertical bars. Means with different letters indicate statistical significance with *p* < 0.05 (a, b); means with at least one common letter are not significantly different (ab), colors of the letters refer to the different treatments. TEER = transepithelial electrical resistance (Ω·cm^2^); CTR = control group; OA + NIC 200 = treated group with 200 ppm of OA + NIC, composed from 260 µM of citric acid, 298 µM of sorbic acid, 23 µM of thymol, and 13 µM of vanillin; OA + NIC 1000 = treated group with 1000 ppm of OA + NIC, composed from 1300 µM of citric acid, 1490 µM of sorbic acid, 110 µM of thymol, and 70 µM of vanillin. (**a**) Cells challenged at day 0 of the study. (**b**) Cell challenged at day 14 of the study.

**Figure 9:**
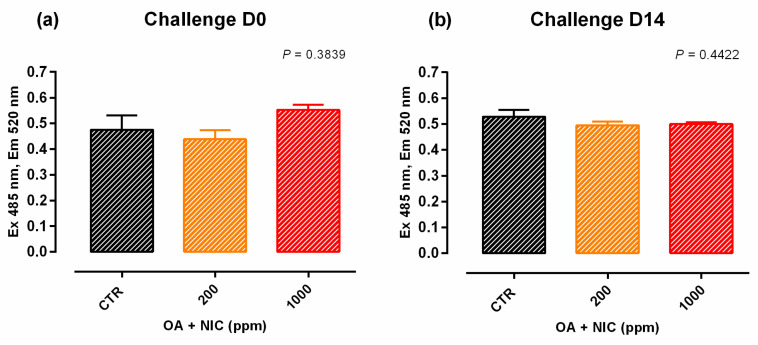
PCP of Caco-2 cells after 15 days of treatment with OA + NIC and subjected to an inflammatory challenge. Data in the graph are means (*n* = 6) ± SEM represented by vertical bars. Means with different letters indicate statistical significance with *p* < 0.05 (a, b); means with at least one common letter are not significantly different (ab). PCP = paracellular permeability (excitation 485 nm, emission 520 nm). CTR = control group; OA + NIC 200 = treated group with 200 ppm of OA + NIC, composed from 260 µM of citric acid, 298 µM of sorbic acid, 23 µM of thymol, and 13 µM of vanillin; OA + NIC 1000 = treated group with 1000 ppm of OA + NIC, composed from 1300 µM of citric acid, 1490 µM of sorbic acid, 110 µM of thymol, and 70 µM of vanillin. (**a**) Cells challenged at day 0 of the study. (**b**) Cell challenged at day 14 of the study.

**Figure 10:**
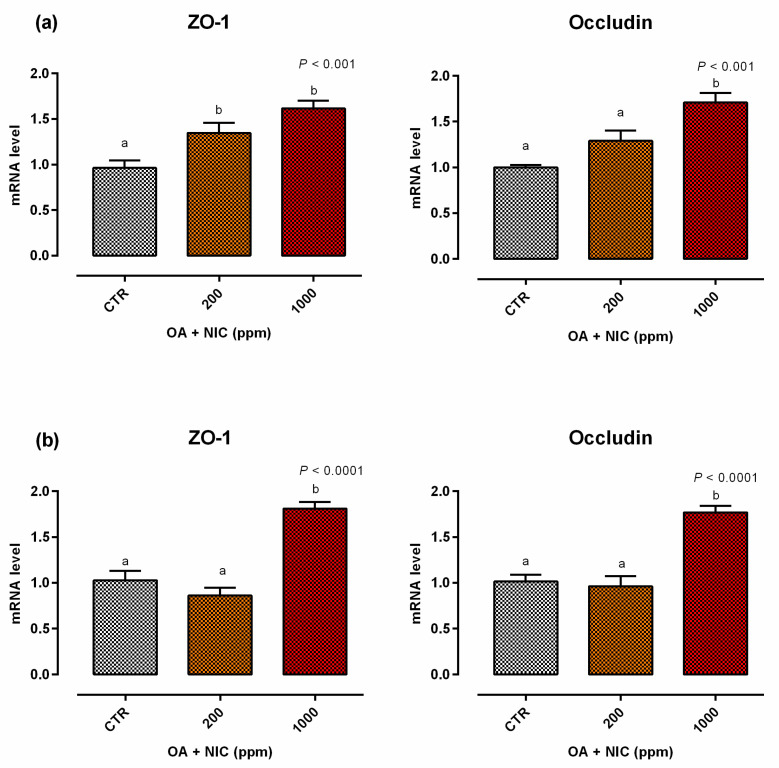
Gene expression in Caco-2 cells after 15 days of treatment with OA + NIC and subjected to an inflammatory challenge. Values are least square means (*n* = 6) ± SEM represented by vertical bars. A modification of the 2^–ΔΔCT^ method [16] was used to analyze the relative expression (fold changes), calculated relative to the control group. Means with different letters indicate statistical significance with *p* < 0.05 (a, b); means with at least one common letter are not significantly different (ab). CTR = control group; OA + NIC 200 = treated group with 200 ppm of OA + NIC, composed from 260 µM of citric acid, 298 µM of sorbic acid, 23 µM of thymol, and 13 µM of vanillin; OA + NIC 1000 = treated group with 1000 ppm of OA + NIC, composed from 1300 µM of citric acid, 1490 µM of sorbic acid, 110 µM of thymol, and 70 µM of vanillin; ZO-1 = zonula occludens. (**a**) Cells challenged at day 0 of the study. (**b**) Cell challenged at day 14 of the study.

**Figure 11:**
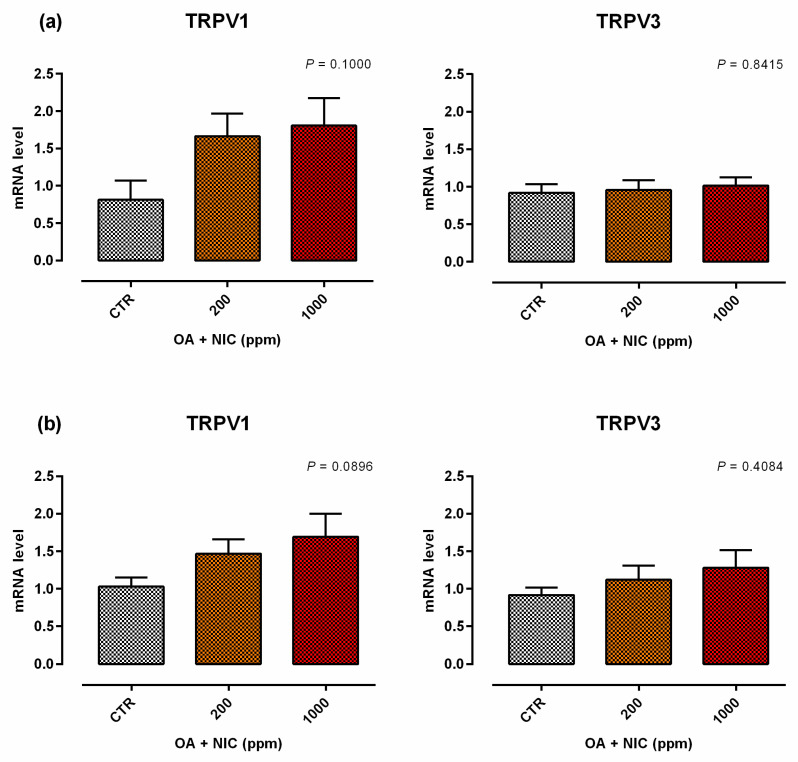
Gene expression in Caco-2 cells after 15 days of treatment with OA + NIC and subjected to an inflammatory challenge. Values are least square means (*n* = 6) ± SEM represented by vertical bars. A modification of the 2^–ΔΔCT^ method [16] was used to analyze the relative expression (fold changes), calculated relative to the control group. Means with different letters indicate statistical significance with *p* < 0.05 (a, b); means with at least one common letter are not significantly different (ab). CTR = control group; OA + NIC 200 = treated group with 200 ppm of OA + NIC, composed by 260 µM of citric acid, 298 µM of sorbic acid, 23 µM of thymol, and 13 µM of vanillin; OA + NIC 1000 = treated group with 1000 ppm of OA + NIC, composed by 1300 µM of citric acid, 1490 µM of sorbic acid, 110 µM of thymol, and 70 µM of vanillin; TRPV1 = Transient Receptor Potential Vanilloid 1; TRPV3 = Transient Receptor Potential Vanilloid 3. (**a**) Cells challenged at day 0 of the study. (**b**) Cell challenged at day 14 of the study.

When the inflammatory challenge was performed on day 0, TEER was increased by the supplementation of OA + NIC 1000 immediately within 24 h from the challenge, compared to CTR, and then constantly throughout all the study. Cells treated with OA + NIC 200 showed a significant increase of the TEER compared to CTR at days 4, 7, 9, 12, and 15 (Figure 8). No variations in PCP were recorded (Figure 9). mRNA level of ZO-1 was significantly increased in both the supplemented groups, while occludin resulted upregulated only in OA + NIC 1000 (Figure 10). A trend of increase in mRNA levels of TRPV1 was reported (*p* = 0.10), while TRPV3 showed no difference between CTR and treated groups (Figure 11).

#### 2.2.2. Preventive Approach

When the inflammatory challenge was performed on day 14, TEER was increased by OA + NIC 200 starting from day 2 and the increment remained significant until the challenge. Cells pre-treated with OA + NIC 1000 showed an increase in TEER at days 5, 9 and 14 before the challenge. After the challenge, TEER remained significantly higher in the cells treated with OA + NIC 1000 compared to CTR (Figure 8). No variations in PCP were recorded (Figure 9). An upregulation of both ZO-1 and occludin was recorded for cells receiving the pre-treatment with OA + NIC 1000 (Figure 10). Moreover, TRPV1 was tendentially increased by the treatments (*p =* 0.08), while TRPV3 showed no difference, compared to CTR (Figure 11).

## 3. Discussion

Organic acids and NIC are widely used as feed additives in animal nutrition because of their beneficial effects on growth performance and intestinal morphology, as well as their antibacterial, antioxidant and anti-inflammatory properties [10,11]. This study focused on the activity of selected OA, citric and sorbic acid, and NIC, thymol and vanillin on human Caco-2 intestinal cell line. First, the aim was to assess the safety and efficacy of these molecules tested individually. Second, we wanted to verify a possible synergistic effect, given by the combination of these molecules and last, we wanted to investigate the effects of the blend of OA and NIC before and after an inflammatory challenge. A previous study conducted by our research group found a reduction in mRNA level of inflammatory cytokines, together with a decrease in paracellular permeability in piglets fed OA + NIC [13]. As OA and NIC are mostly known for their ability to positively modulate gut microbial population [17,18,19,20,21,22,23,24], in vivo results might be mediated by the immunomodulatory and epigenetic interplay between host and intestinal microflora [25]. As a consequence, we wanted to verify the effect of each compound directly on the epithelium.

The most striking result is that each of the molecules tested individually had little or no effect on Caco-2, whereas when they were combined together they improved epithelial barrier by increasing TEER and TJ components gene expression. Interestingly, the OA + NIC blend at 200 ppm, corresponding to the lowest dose of the individual compounds, had a minor effect on the measured parameters. Whereas, the blend at 1000 ppm consistently improved TEER and ZO-1 expression despite the individual compounds at much lower doses started to have some cytotoxic effect. We previously reported the synergy of OA and NIC, in terms of antibacterial action [26] and intestinal mucosa functionality in weaned pigs [13]. Additionally, it was demonstrated a positive effects on TEER on Caco-2 cells [13]. In the present study, we wanted to explain the role of the single components of the blend, and moreover, the distinct responses of OA + NIC against an inflammatory challenge. Caco-2 are a validated model to study intestinal function as the cells are able to polarize and form the brush border. Once differentiated they express a phenotype common to both enterocytes and colonocytes, and they are a useful tool for absorption and intestinal transport studies [15]. Moreover, Caco-2 preserve many characteristics of the live tissue they are derived from, as they are able to synthetize enzymes and cytokines [15]. Nevertheless, this model has limitations, including that it lacks a lamina propria, and it does not possess the underlying functions that a more complex organism is able to perform, including, but not limited to, blood perfusion and circulation, as well as an immune response. Citric acid is an intermediate of the Krebs cycle and might have a role in cell metabolism [27]. Although, in vitro testing is a strong chelator and can interfere with calcium transport, thereby, affecting TEER and TJ expression [28,29]. Sorbic acid is a medium chain unsaturated fatty acid that is metabolized via beta oxidation but can have a role in the signaling of insulin-like growth factor (IGF) pathway [30]. In the current study, citric and sorbic acid exerted a slight cytotoxic effect at high doses when used individually, no negative effects were reported when combined with thymol and vanillin. Thymol and vanillin are botanical compounds that have in vitro and in vivo anti-inflammatory and anti-oxidant activity and are widely used in animal nutrition [11,31]. The effects of thymol on intestinal secretory activity were studied ex vivo by Boudry and Perrier [32] and Michiels et al. [33] demonstrated that thymol induces ion secretions in the small intestine via the nervous system. As electrolytes and water secretion is usually a means to eliminate bacterial toxins from the lumen, thymol may plays a role in the defense of the organism and consequently affects the barrier function.

Although, the present study was limited to the analysis of only few aspects of barrier function, it is interesting to note how the selected OA and NIC conferred a stronger trait of resistance to polarized Caco-2 under healthy or challenged conditions. The measurement of TEER reflects the ionic conductance of the paracellular pathway, while PCP indicates the paracellular dextran flow, as well as the pore size of the TJ [34]. Despite the absence of variation on PCP mediated by OA + NIC, the blend showed ameliorative effects, in terms of TEER and TJ, both in normal and challenging conditions, most likely driven by thymol and vanillin. In fact vanillin, beside anti-inflammatory and anti-microbial properties, is also potentially targeting TRPV channel. These TRP channels are non-selective cation channels, expressed on the cell membrane, and exhibit variable permeability ratios for Ca^2+^ versus Na^+^. They mediate many functions among which divalent cation flux [35]. The same potential to act as possible ligand of these channels is empowered by thymol [35]. As the cation flux regulation is essential during a challenge induced by toxins secreted by *Escherichia coli,* for instance, the contribution of these compounds in maintaining intestinal homeostasis can be relevant. Furthermore, the modulation of these channels from OA + NIC, with an increased expression of TRPV1 and a reduction of TRPV3, support the hypothesis of the anti-inflammatory potential of the mixture. In fact, TRPV1 and TRPV3 are known for their anti-inflammatory [36], and pro-inflammatory [5] action, respectively. Moreover, the cytokine cascade induced by the host inflammatory response has a direct effect on TJ proteins. In fact, it is well-documented, that IFN-γ and TNF-α can cause a direct disruption of ZO-1 and occludins [37] so that the increased expression of these proteins by Caco-2 cells, during an inflammatory challenge, might be the direct consequence of the beneficial and anti-inflammatory effect given by the mix of OA + NIC.

Interestingly, this experiment resulted from the dualistic approach, either preventive or therapeutic. In the first case, the application of the mix of OA + NIC for 14 days before the challenge allowed the cells to be more prepared to the damages deriving by the inflammatory challenge. In particular, the preventive administration of OA + NIC led the cells to deal with the challenging stimuli, avoiding the fall in TEER and TJ gene expression correlated with an inflammatory challenge. Whereas in the second case, the application of the OA + NIC mix right at the time of the challenge allowed the cells to recover their functionality properties already after few days, with a re-establishment or an improvement in the parameters evaluated. If translated in vivo, these results could constitute the starting point of a possible use of these compounds either as to imprint the epithelium and the mucosa of the newly weaned animal, to make it more resistant to future challenges, or as non-antibiotic molecules to support conventional medical treatments and therapies that are commonly used at the onset of enteric disorders.

## 4. Materials and Methods

### 4.1. Chemicals and Reagent

Cell culture reagents and chemicals were provided by Sigma-Aldrich (Milan, Italy), unless something different specified. All chemicals were analytical grade: citric acid (99%), thymol (≥98.5%), and vanillin (99%) were obtained from Sigma-Aldrich (Milan, Italy), sorbic acid (99%) was purchased from Chem-Lab (Chem-Lab NV, Zedelgem, Belgium).

### 4.2. Cell line and Culture Conditions

The human colon adenocarcinoma cell line (Caco-2) was obtained from DSMZ (DSMZ-German Collection of Microorganisms and Cell Cultures, Leibniz Institute, Germany). Caco-2 cells were maintained at 37 °C, in an atmosphere containing 5% CO_2_ at 95% relative humidity, in a medium (*basal medium*) consisting of DMEM supplemented with 10% fetal bovine serum (FBS), 1% non-essential amino acids (NEAA), 1% penicillin/streptomycin (P/S) and 1% L-Glutamine. Then Caco-2 cells were cultured in two different systems based on the analysis subsequently performed: (1) 96-well plates, where cells were seeded at density of 1.5 × 10^4^ cells/well and cultured in basal medium; (2) 24 well transwell polyethylene terephthalate inserts (0.4 µm pore; Corning, Amsterdam, Nederland), where cells were seeded at a density of 5 × 10^4^ cells/transwell.

### 4.3. Viability Assay on Caco-2

Screening of cell viability was assessed with PrestoBlue reagent (Invitrogen, ThermoFisher Scientific, Milan, Italy). Briefly, Caco-2 cells were seeded onto 96-well plates and cultured in basal medium. When cells reached the confluence, the medium was supplemented with specific treatments and their relative control. The treated cells were tested for their viability 24 h and 7 days after each treatment, following the manufacturer instructions. Fluorescence values were recorded with Varioskan LUX (Thermofisher Scientific). The % of cell viability was calculated by the following formula, where *mean fluo* is the fluorescence of cells with substances and *mean control* represent the fluorescence of the control group (adapted from Sharma [38]):

Cell Viability % = Mean Fluo/Mean control × 100.(1)

### 4.4. Measurement of Trans-Epithelial Electrical Resistance (TEER)

Cells were seeded onto 24 transwell and TEER was measured every other day after cells reached confluence, using an epithelial tissue voltohmmeter (Millicell ERS-2, Merk, Merckmillipore, Germany). The experiment started 28 days after the seeding on filters once TEER values were stable (~150 Ω∙cm^2^), then cells were treated with the substances for 15 days. During the treatment TEER was measured at 0, 2, 4, 7, 9, 11, 14 and 15 days.

### 4.5. Measurement of Paracellular Permeability (PCP)

Cells were seeded onto 24 transwell and PCP was recorded as apical-to-basolateral passage of dextran. PCP was measured on day 15 by applying fluorescein isothiocyanate–dextran (FD4) (100 mg/mL) on the apical well of the transwell and collecting basal media after 24 h for measurements of FD4 fluorescence. Fluorescence values for PCP were recorded with Varioskan™ LUX (Thermofisher Scientific, Milan, Italy).

### 4.6. Gene Expression Analysis

At the end of the experiments onto 24 transwell, cells were collected, snap-frozen in liquid nitrogen and stored at −80 °C in lysis buffer (Macherey-Nagel, Düren, Germany) until gene expression analysis. RNA was isolated using NucleoSpin RNA Kit (Macherey-Nagel) and genomic DNA contamination was removed with deoxyribonuclease (rDNA RNase-Free; Macherey-Nagel), according to the manufacturer’s instruction. RNA yield and quality were determined spectrophotometrically using A260 and A280 nm measurements (Microvolume Mode with SmartPath Technology, Denovix). A total of 450 ng of RNA was reverse-transcribed for each sample with iScript cDNA Synthesis Kit (Bio-Rad Laboratories Inc., Hercules, CA, USA), according to the manufacturer’s instructions. Real-time PCR was performed using CFX96 Touch Real-Time PCR Detection System (Bio-Rad) and iTaq Universal SYBR Green Supermix (Bio-Rad). Gene expression was normalized using two reference genes, such as ribosomal protein lateral stalk subunit P0 (RPLP0) and glyceraldehyde-3-phosphate dehydrogenase (GAPDH). A modification of the 2^–ΔΔCT^ method [16] was used to analyze the relative expression (fold changes), calculated relative to the control group. The sequences, expected product length, accession number in the GenBank database and reference primers are shown in Table 1. Primers were obtained from Sigma-Aldrich.

### 4.7. Experiment 1—Dose Response

The aim was to investigate whether OA and NIC, alone or combined in a blend (OA + NIC), have an impact on intestinal epithelial barrier function. A viability assay in 96-well plates was performed to determine the response of Caco-2 to increasing concentrations of any single bioactive, or the blend OA + NIC. Concentrations of the substances were selected to meet the composition of a product used in our previous research study [13]. Citric acid was tested at 130, 260, 391, 651, 911, 1172, and 1300 µM; sorbic acid was tested at 149, 298, 447, 745, 1040, 1340, and 1490 µM; concentrations of thymol tested were 11, 23, 35, 58, 81, 104, and 110 µM, while vanillin was tested at 7, 13, 20, 35, 46, 59, and 70 µM. OA + NIC was evaluated at the following concentrations: 100, 200, 300, 500, 700, 900, and 1000 ppm (consistent with the inclusions of the single compounds, as reported in Table 2).

Concerning the effect of the substances on the intestinal functionality, on day 28 cells on transwell filters were divided in groups and treated with different doses of citric acid, sorbic acid, thymol, vanillin, or a blend of all these molecules (OA + NIC). Citric acid was tested at 260, 455, and 651 µM, sorbic acid was tested at 298, 521, and 745 µM, thymol was tested at 23, 40, and 58 µM, and vanillin was tested at 13, 23, and 35 µM. Concerning the blend, cells were treated with OA + NIC at 200 or 1000 ppm [13] (subsequently named as OA + NIC 200 and OA + NIC 1000). OA + NIC 200 is composed from 260 µM of citric acid, 298 µM of sorbic acid, 23 µM of thymol, and 13 µM of vanillin, while OA + NIC 1000 is composed from 1300 µM of citric acid, 1490 µM of sorbic acid, 110 µM of thymol, and 70 µM of vanillin. At the end of the experiments cells were collected for gene expression analysis.

### 4.8. Experiment 2—Inflammatory Challenge

The purpose was to assess the preventive or ameliorative potential of the blend OA + NIC against an inflammatory challenge. Inflammatory challenge was induced for 24 h and consisted in the exposure to a cocktail of pro-inflammatory cytokines such as IL-1β (25 ng/mL), TNF-α (50 ng/mL), IFN-γ (50 ng/mL), and LPS from *E. coli* O55:B5 (10 ug/mL) (adapted from Van de Walle [44]).

Concerning the effect on the intestinal functionality, on day 28 Caco-2 cells on filters were divided into 3 groups: control (CTR), OA + NIC 200 and OA + NIC 1000. The challenge was performed at 2 different time-point to evaluate the enhancing or preventive properties of the blend.

1. *Therapeutic approach*: the challenge was performed at day 0, along with treatments, and stopped after 24 h, then cells continued to receive the different treatments for 15 days.
2. *Preventing approach*: the challenge was performed at day 14, after treatments, and stopped after 24 h. Cells received the different treatments for a total of 15 days.

At the end of the experiments cells were collected for gene expression analysis.

### 4.9. Statistical Analysis

Data were analyzed using GraphPad Prism (GraphPad Software, Inc., La Jolla, CA, USA). ANOVA repeated measures was used for TEER. One-way ANOVA, followed by Bonferroni and Tukey’s post-test was used for PCP, gene expression, and viability assay. The experimental unit was the well (*n* = 6). Differences were considered significant at *p* < 0.05, trends at 0.05 < *p* ≤ 0.10.

## 5. Conclusions

To conclude, OA and NIC have different chemical and biological properties that are best exploited when combined, as they address a wide range of molecular targets. By modulating the antioxidant and anti-inflammatory status [40,41], cell metabolism [23,26] and ion transport [42,43] these molecules have the potential to ameliorate intestinal barrier function under normal or challenged conditions in vitro. This would justify their use in vivo as feed additives, although more studies need to be performed to assess the mode of action of these bioactive compounds, in vivo. In fact, in vitro systems are not predictive of the response of the immune system, nor of digestion and absorption, as well as they do not take into consideration the complicated interplay between the host and microflora.

Nevertheless, this study highlighted some specific features of this combination of sorbic, citric acid and thymol and vanillin in preventing and ameliorating intestinal epithelium loss of integrity that might be at the core of the efficacy of these compounds in vivo.

## Figures and Tables

**Table 1:** Primers used for gene expression analysis.

Gene	Primer Sequence (F and R) 5′ → 3′	Product Length (bp)	Accession N.	Reference
ZO-1	F: CGGGACTGTTGGTATTGGCTAGA R: GGCCAGGGCCATAGTAAAGTTTG	184	NM_001301025.3	[39]
OCCL	F: TCCTATAAATCCACGCCGGTTC R: CTCAAAGTTACCACCGCTGCTG	105	NM_001205254.2	[39]
TRPV1	F: GACCACCTGGAACACCAACG R: TGAGCAGACTGCCTATCTCG	177	NM_080704.3	[40]
TRPV3	F: GAGCAGATTCCGGATGGGA R: CCGCAAACACAGTCGGAAA	64	NM_001258205.1	[41]
RPLP0	F: GCAATGTTGCCAGTGTCTG R: GCCTTGACCTTTTCAGCAA	142	NM_001002.3	[42]
GAPDH	F: TGCACCACCAACTGCTTAGC R: GGCATGGACTGTGGTCATGAG	87	NM_02046	[43]

F = forward; R = reverse; ZO-1 = zonula occludens-1; OCCL = occludin; TRPV1 = Transient Receptor Potential Vanilloid 1; TRPV3 = Transient Receptor Potential Vanilloid 3; OR1G1 = Olfactory Receptor Family 1 Subfamily G Member 1; RPLP0 = Ribosomal Protein Lateral Stalk Subunit P0; GAPDH = Glyceraldehyde 3-phosphate dehydrogenase.

**Table 2:** Composition of the blend OA + NIC at the concentration tested for the viability assay. The inclusions of the single compounds in each row correspond to the amount of the molecules in the mixture (OA + NIC).

OA + NIC (ppm)	Citric Acid (µM)	Sorbic Acid (µM)	Thymol (µM)	Vanillin (µM)
100	130	149	11	7
200	260	298	23	13
300	391	447	35	20
500	651	745	58	35
700	991	1040	81	46
900	1172	1340	104	59
1000	1300	1490	110	70

## Supplementary Materials

The following are available online at https://www.mdpi.com/1420-3049/25/18/4296/s1: Figure S1: Viability of Caco-2 cells treated for 24 h and 7 days with incremental doses of single OA and NIC, Figure S2: Viability of Caco-2 cells treated for 24 h and 7 days with incremental doses of the blend of OA + NIC.
