# Supplementary material for: Nature-Identical Compounds and Organic Acids Ameliorate and Prevent the Damages Induced by an Inflammatory Challenge in Caco-2 Cell Culture
Source: Molecules. 2020 Sep 19;25(18):4296. doi: 10.3390/molecules25184296 (PMC7570934; doi:10.3390/molecules25184296)
Supplement: Supplementary file 1 [file molecules-25-04296-s001.pdf]

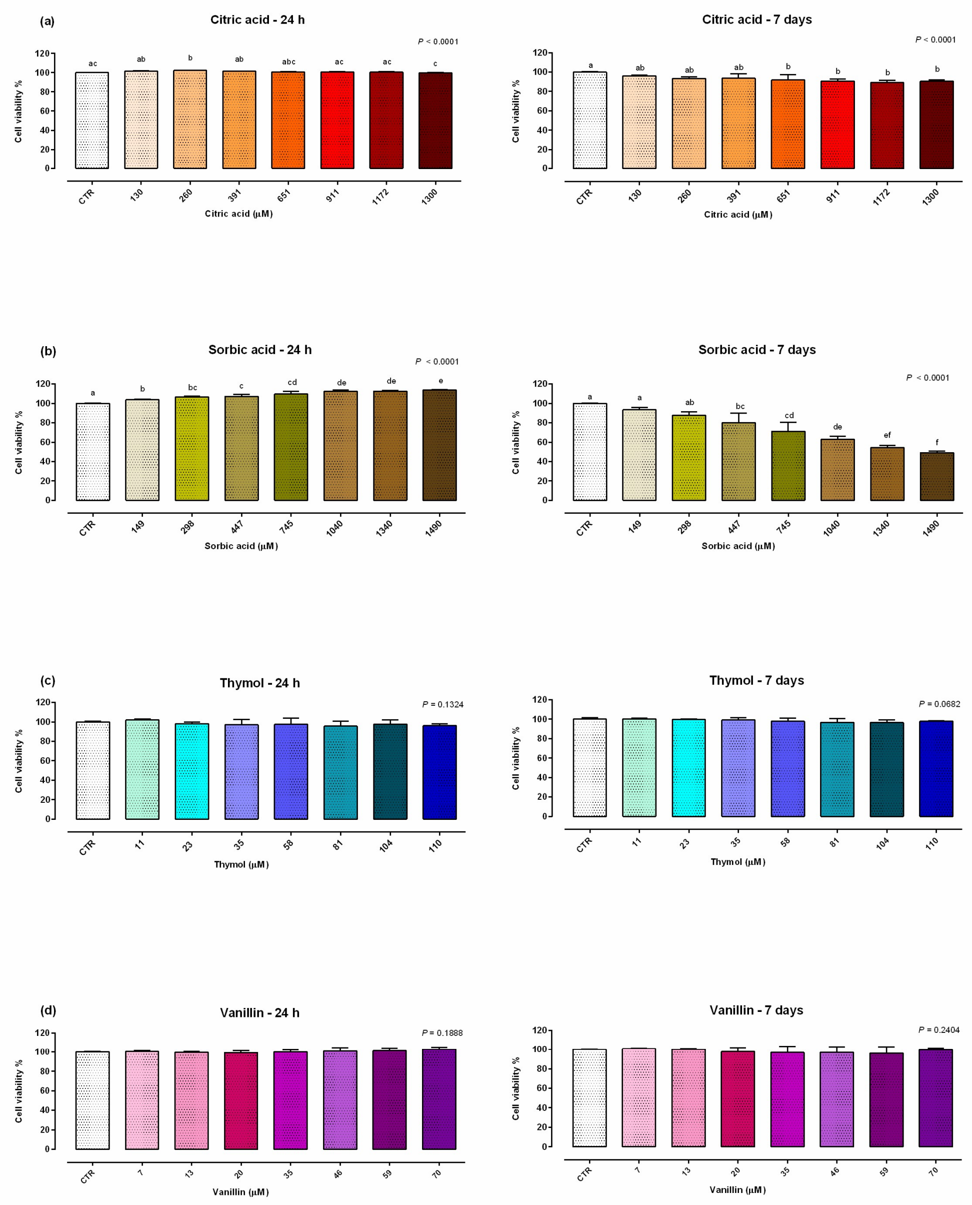

Figure S1. Viability of Caco-2 cells treated for 24 hours and 7 days with incremental doses of single OA and NIC. Data in the graph are means (n = 6) ± SEM represented by vertical bars. Means with different letters indicate statistical significance with $p < 0.05$; means with at least one common letter are not significantly different. **(a)** Cells treated with incremental doses of citric acid. CTR = control group; 130 = treated group with 130 μM of citric acid; 260 = treated group with 260 μM of citric acid; 391 = treated group with 391 μM of citric acid; 651 = treated group with 651 μM of citric acid; 911 = treated group with 911 μM of citric acid; 1172 = treated group with 1172 μM of citric acid; 1300 = treated group with 1300 μM of citric acid. **(b)** Cells treated with incremental doses of sorbic acid. CTR = control group; 149 = treated group with 149 μM of sorbic acid; 298 = treated group with 298 μM of sorbic acid; 447 = treated group with 447 μM of sorbic acid; 745 = treated group with 745 μM of sorbic acid; 1040 = treated group with 1040 μM of sorbic acid; 1340 = treated group with 1340 μM of sorbic acid; 1490 = treated group with 1490 μM of sorbic acid. **(c)** Cells treated with incremental doses of thymol. CTR = control group; 11 = treated group with 11 μM of thymol; 23 = treated group with 23 μM of thymol; 35 = treated group with 35 μM of thymol; 58 = treated group with 58 μM of thymol; 81 = treated group with 81 μM of thymol; 104 = treated group with 104 μM of thymol; 110 = treated group with 110 μM of thymol. **(d)** Cells treated with incremental doses of vanillin. CTR = control group; 7 =

treated group with 7 μM of vanillin; 13 = treated group with 13 μM of vanillin; 20 = treated group with 20 μM of vanillin; 35 = treated group with 35 μM of vanillin; 46 = treated group with 46 μM of vanillin; 59 = treated group with 59 μM of vanillin; 70 = treated group with 70 μM of vanillin.

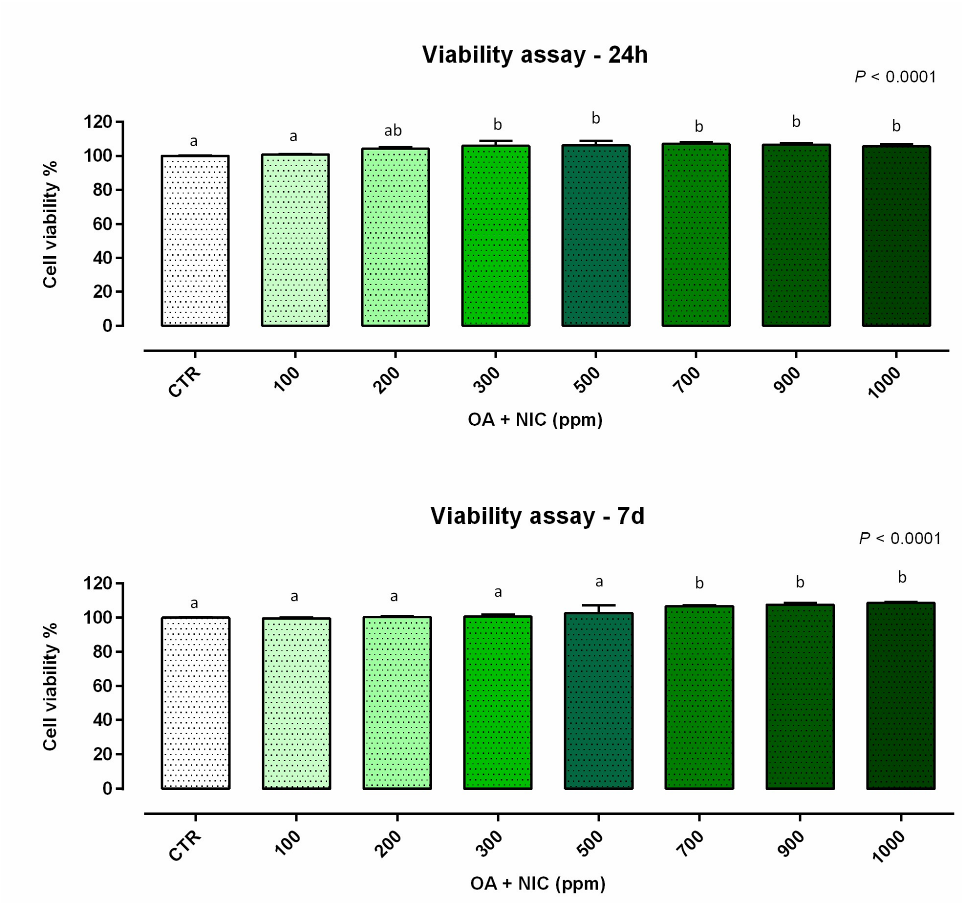

Figure S2. Viability of Caco-2 cells treated for 24 hours and 7 days with incremental doses of the blend of OA + NIC. Data in the graph are means (n = 6) ± SEM represented by vertical bars. Means with different letters indicate statistical significance with $p < 0.05$; means with at least one common letter are not significantly different. CTR = control group; 100 = treated group with 100 ppm of the blend; 200 = treated group with 200 ppm of the blend; 300 = treated group with 300 ppm of the blend; 500 = treated group with 500 ppm of the blend; 700 = treated group with 700 ppm of the blend; 900 = treated group with 900 ppm of the blend; 1000 = treated group with 1000 ppm of the blend.
